# The Bacterial (*Vibrio alginolyticus*) Production of Tetrodotoxin in the Ribbon Worm *Lineus longissimus*—Just a False Positive?

**DOI:** 10.3390/md14040063

**Published:** 2016-03-25

**Authors:** Malin Strand, Martin Hedström, Henrik Seth, Eric G. McEvoy, Erik Jacobsson, Ulf Göransson, Håkan S. Andersson, Per Sundberg

**Affiliations:** 1 Swedish Species Information Centre, Swedish University of Agricultural Sciences, 75007 Uppsala, Sweden; 2 Division of Biotechnology, Lund University, 22100 Lund, Sweden; martin.hedstrom@biotek.lu.se; 3 Department of Biological and Environmental Sciences, University of Gothenburg, 40530 Gothenburg, Sweden; henrik.seth@bioenv.gu.se (H.S.); per.sundberg@bioenv.gu.se (P.S.); 4 Department of Neuroscience and Physiology, Sahlgrenska Academy, University of Gothenburg, 40530 Gothenburg, Sweden; 5 School of Natural Sciences and Psychology, Liverpool John Moores University, Liverpool L32AJ, UK; eric.mcevoy@sky.com; 6 Division of Pharmacognosy, Department of Medicinal Chemistry, Uppsala University, 75237 Uppsala, Sweden; erik.jacobsson@fkog.uu.se (E.J.); ulf.goransson@fkog.uu.se (U.G.); 7 Linnaeus University Centre for Biomaterials Chemistry, Department of Chemistry and Biomedical Sciences, Linnaeus University, 39234 Kalmar, Sweden

**Keywords:** LC-MS, mucus, axonal conductance, *Vibrio*, nemertean, tetrodotoxin

## Abstract

We test previous claims that the bacteria *Vibrio alginolyticus* produces tetrodotoxin (TTX) when living in symbiosis with the nemertean *Lineus longissimus* by a setup with bacteria cultivation for TTX production. Toxicity experiments on the shore crab, *Carcinus maenas*, demonstrated the presence of a paralytic toxin, but evidence from LC-MS and electrophysiological measurements of voltage-gated sodium channel–dependent nerve conductance in male Wistar rat tissue showed conclusively that this effect did not originate from TTX. However, a compound of similar molecular weight was found, albeit apparently non-toxic, and with different LC retention time and MS/MS fragmentation pattern than those of TTX. We conclude that *C. maenas* paralysis and death likely emanate from a compound <5 kDa, and via a different mechanism of action than that of TTX. The similarity in mass between TTX and the *Vibrio*-produced low-molecular-weight, non-toxic compound invokes that thorough analysis is required when assessing TTX production. Based on our findings, we suggest that re-examination of some published claims of TTX production may be warranted.

## 1. Introduction

Tetrodotoxin (TTX) is a highly potent neurotoxin found in a number of organisms such as puffer fish, salamanders, frogs, octopi, flatworms, crustaceans and nemerteans [1]. The toxicity stems from the ability of TTX to block, by binding to the neurotoxin receptor site outside of the pore, several voltage-gated sodium channels, so-called Na_V_ or VGSC. The Na_V_ channels exist in several distinct isoforms with a specific tissue distribution and a variable sensitivity to TTX [2,3], including a few TTX-insensitive isoforms found in cardiac myocytes and dorsal root ganglia (Na_V1.5_, Na_V1.8_ and Na_V1.9_) [4,5]. The biogenic origin of TTX is a subject of debate, and the biosynthesis is yet to be elucidated [6]. At least in the case of marine organisms, the presence of TTX has been linked to bacteria, and several *Vibrio* species are reported in association with TTX production in a variety of marine vertebrate and invertebrate species. It has been suggested that *Vibrio* spp., in a symbiotic relationship with the host [7], could be involved in the biosynthesis of TTX.

Nemerteans rely upon a variety of toxins for their defense, such as anabasine and pyridyl alcohols, but toxins are also used by these predators as part of their hunting strategy [8]. The presence of TTX in certain Anopla species (*Lineus fuscoviridis* and *Tubulanus punctatus*) was first reported by Miyazawa *et al.* in 1988 [9]. Since then, the presence of TTX and related compounds has been described in a number of species [10,11,12,13,14,15,16,17] (Table 1). The production of TTX in nemerteans has been proposed to rely on bacteria, and McEvoy *et al.* suggested the presence of *V. alginolyticus*–like strains, found in 10 nemertean species, as the source of TTX [18]. Moreover, Magarlamov *et al.* recently extracted a novel Bacillus strain from *Cephalothrix simula*, and indicated the presence of TTX on the bacterial cell wall through immunofluorescence staining [17]. However, unambiguous proof of where TTX is produced and if the production is dependent on symbiotic bacteria or not is still lacking. Notably, also, the biosynthetic pathway of TTX is unknown.

In the present study, we follow the strategy of Carroll *et al.* [10] to investigate whether *Vibrio alginolyticus* isolates from the mucus of *L. longissimus* produce TTX. Toxicity is established using shore crab assay, and toxic compounds are assayed using LC-MS analysis and by studying the inhibitory effects of the potential TTX on basal axonal conductance in rat hippocampal slices, a response dependent on voltage-gated sodium channels.

## 2. Results and Discussion

The mucus released by some nemerteans has been reported to contain several bioactive compounds, of which TTX is suggested to be one of the most potent. The heteronemertean *L. longissimus* releases large amounts of mucus when physically challenged. In the current work, that mucus was collected when released into a small volume of surrounding sea water. The filtered collection was then tested *in vivo* on shore crabs (*Carcinus maenas*). The shore crab assay provides means to study the effect on a crustacean species, which are both natural prey and potential predators of many nemertean species. The quick pharmacological response of the mucus *in vivo* is analogous to that of TTX. In our assay, TTX caused contraction and paralysis within 20 s when the shore crab was subjected to a 2 μg dose. This led us to hypothesize that the mucus of *L. longissimus* contains TTX, as has been reported for other nemertean species. Injections of lower doses gave a delayed onset and partial paralysis, or at even lower levels, no visible effect at all. *Vibrio* bacteria have been suggested to be the common denominator for a range of TTX-containing organisms, including several species of the genera *Lineus* [9,10,16]. Hence, it was reasonable to test (i) if *Vibrio* bacteria are present on the surface or in the mucus of *L. longissimus*; and (ii) if we could isolate and cultivate *Vibrio* bacteria with TTX-like activity.

### 2.1. Establishment and Cultivation of Vibrio Alginolyticus

Most plates generated 4–10 colonies with a maximum diameter of 2.5 mm. Each colony was sequenced (16S rRNA) and identification was performed based on 16S rRNA sequence homology [19] via standard nucleotide blast in Genbank. It may be worth noting that although this is the preferred method, it has been claimed to compare poorly to other identification methods [20]. Based on this analysis, approximately 80% of all colonies could be identified as *Vibrio alginolyticus*. As this particular species is one of the *Vibrio* bacteria that has been implicated in TTX production [7,21], it was chosen for the establishment of a large-scale culture; selected colonies from one experiment were thus combined and transferred to one single culture in a liquid medium rich in nutrients. *V.* *alginolyticus* grew quickly in the rich nutrient broth media, reaching the lag phase within 48 h. The liquid medium was sampled at different time points and harvested after 72 h. Supernatants were then collected by centrifugation and filtration and subjected to analysis by MS via direct infusion, LC-MS and shore crab assay.

### 2.2. Chemical Analyses for the Detection of TTX

No TTX could be detected using MS analysis via direct infusion of the harvested medium supernatants. To circumvent the drawbacks of MS analysis without separation, such as ion suppression, an LC-MS method was established to detect and quantify TTX. However, this method could neither detect TTX at the different time points, nor could any TTX-like activity be detected using the shore crab assay. We then tested the hypothesis that TTX production is triggered by the presence of *L. longissimus* mucus. Therefore, in a following set of experiments, mucus, sterile filtrated or autoclaved, was added to *V. alginolyticus* cultures in separate experiments in an attempt to elicit the production. However, no change in activity or chemistry of the supernatant could be detected under any conditions (data not shown).

**Figure 1:**
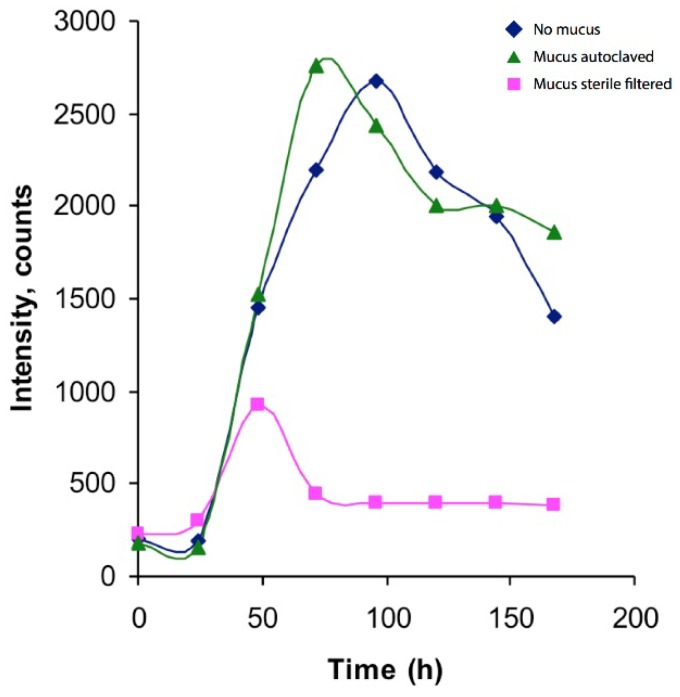
Production profile of *m*/*z* 320.08. Blue line represents *Vibrio* culture without addition of *L. longissimus* mucus, whereas green line represents *Vibrio* culture to which autoclaved mucus has been added, and pink line represents the *Vibrio* culture with sterile filtered mucus added. An increase of the *m*/*z* 320.08 compound is seen over the first 50 (sterile filtered), 80 (autoclaved) and 100 (control) hours, respectively, followed by a decline. The analyte does not appear to originate from the mucus but is rather a product of the *Vibrio* culture or medium. These results originate from duplicate injections of single culture samples.

**Figure 2:**
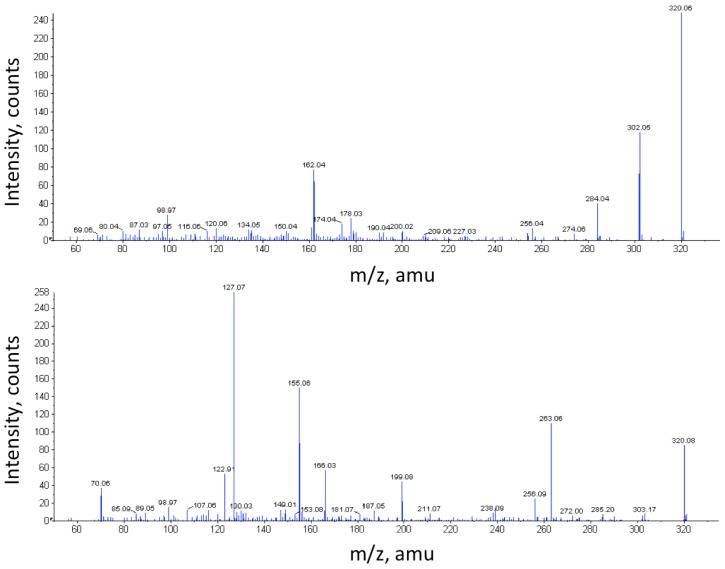
MS/MS spectra of TTX standard and the unknown compound with *m*/*z* 320.08. MS/MS spectra of the TTX standard (320.06; top) and the observed unidentified compound (320.08; bottom).

**Figure 3:**
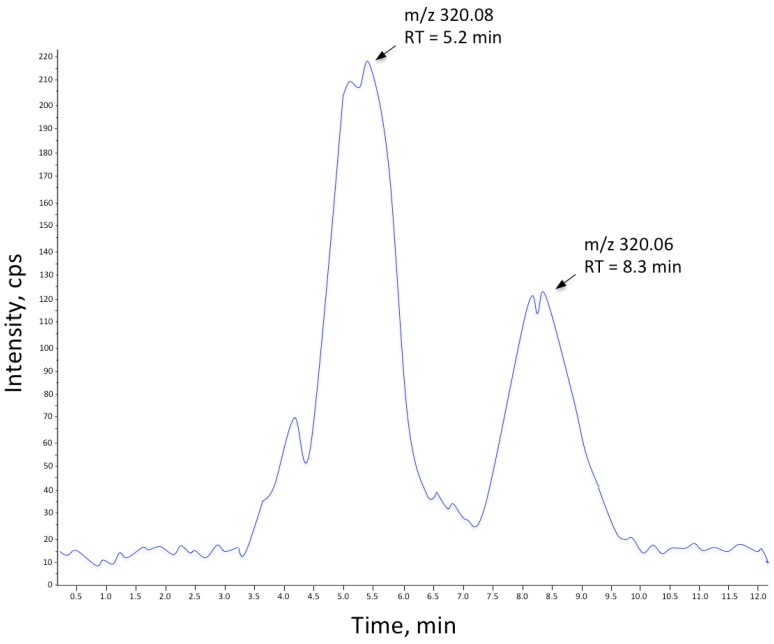
LC trace of the unknown compound with *m*/*z* 320.08 and TTX standard. Total ion count spectra (LC-MS/MS) demonstrating the difference in retention time between TTX (*m*/*z* 320.06; RT 8.3 min) and the unidentified compound (*m*/*z* 320.08; RT 5.2 min).

Notably, a substance with *m*/*z* 320.08 (M + H^+^) could be followed throughout the cultivation of *V. alginolyticus* (Figure 1). This corresponds rather well to the mass of the TTX standard (M + H^+^, *m*/*z* 320.107). However, the MS/MS spectra (Figure 2) display markedly different fragmentation patterns, clearly indicating that the unidentified compound is structurally different from TTX, a conclusion further supported by their differing elution times (Figure 3). It can also be seen that the samples from the control cultivation (with no mucus added) and the samples from the cultivation with autoclaved mucus added showed almost overlapping concentrations of the unknown substance throughout the cultivation. Moreover, upon comparison to the characteristics of known TTX derivatives [22], no relationship is evident. This observation is relevant in light of the observation made by Matsumura [23] pointing to the risk of identifying false positives in the analysis of TTX. It should be mentioned that from the results of our analysis of the TTX standard, an *m*/*z* of 320.06 was detected. Hence, a minor mass calibration error (Δ*m*/*z* 0.046) in the utilized mass spectrometer could be concluded. The relative mass difference of the two compounds (*m*/*z* 320.06 *vs.* *m*/*z* 320.08) should, however, not be affected in this case.

In addition, mucus from living specimens, with proven activity in the shore crab assay, was analyzed using the same LC-MS system, with the same negative result: no TTX could be detected. Then we tried to concentrate compounds in the mucus by passing a large volume (245 mL) of filtrated mucus, as released from the nemertean into the surrounding water, through reversed phase material (C8 modified silica). Although the enrichment method had been tested with positive results using reference TTX, no TTX could be detected in the sample fractions. Also, no activity could be detected in the shore crab assay.

TTX has been reported to bind to plasma proteins [24], and to exclude the possibility that TTX escaped detection as a result of binding to proteins in the mucus, the mucus was mixed with bovine serum albumin (BSA) and fractionated by size exclusion chromatography. No toxic response was achieved for the fractions where BSA eluted; however, activity could still be detected below the molecular separation range of the resin used (<10 kDa). We then switched to affinity chromatography (using boronate-modified resin aimed at compounds containing vicinal diols) coupled online to MS to increase the sensitivity and selectivity of the analysis. However, in that system did we did not manage to detect TTX in the low molecular weight fraction (<10 kDa). When injecting samples of mucus, with and without being spiked with TTX, on the same boronate resin for preparative affinity chromatography, we could again demonstrate that the activity eluted in a different fraction than TTX. From these experiments we could conclude that the activity is mediated by a compound other than TTX itself. Filter fractionation further indicated that the active molecule has a molecular weight of <5 kDa.

So, if the active compound is not TTX although the paralytic effect on shore crab is similar, is the effect mediated through TTX-sensitive ions channels? The most prominent feature of TTX is the capacity to block vertebrate sodium channels [25]. Hence, we tested the activity of the mucus in a rat hippocampal *in vitro* assay.

**Figure 4:**
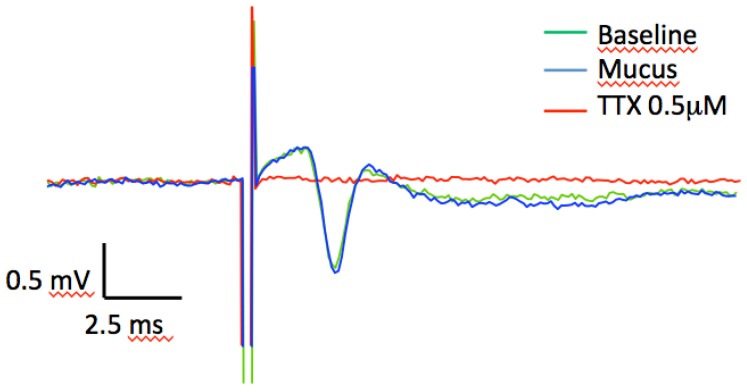
Hippocampal *in vitro* assay. Axonal conductance in Schaffer collaterals of stratum radiatum in rat hippocampal slices. We tested the potency of the nemertean-derived substance in blocking nerve transmission in isolated preparations from the rat hippocampus. Field stimulations of Schaffer collaterals from CA3 pyramidal cells using a tungsten electrode elicit a rapid depolarization and action potential in the stimulated axons, which is recorded as a negative slope (fiber volley) just after the stimulation artifact. This response is critically dependent on voltage-gated sodium channels. Recordings were done in stratum radiatum so as to minimize the slow depolarization that follows the fiber volley, *i.e.*, the excitatory post-synaptic potential (EPSP) that is instead a measurement of synaptic transmission. All experiments were started with baseline recordings of the fiber volley (in green), followed by increasing doses of nemertean toxin (in blue; as described in the methods section). Experiments were then finished by the addition of commercially available TTX (in red).

We recorded the axonal conductance (*i.e.*, fiber volley) from the CA1 stratum radiatum in hippocampal slice preparations from Wistar rat by stimulating the Schaffer collateral fibers. The volley, in contrast to the field excitatory post-synaptic potential (fEPSP), is entirely due to the conductance (*i.e.*, the action potential) over the length of the stimulated axons, and the conductance is critically dependent on the action of TTX-sensitive, voltage-gated sodium channels (Na_V1.1_, Na_V1.2_, Na_V1.3_ and Na_V1.6_). Adding increasing doses (corresponding to 0.5–6.0 µM) of the mucus to the perfusion solution did not inhibit the volley. No inhibitory response was detected at doses that even exceeded a concentration corresponding to a TTX-like effect in the shore crab assay, which are commonly used to inhibit TTX-sensitive channels, by 12 times (Figure 4). As expected, adding 5 µM of commercially available TTX completely blocked the voltage-gated sodium channels and therefore rapidly abolished the volley. Decreasing the TTX concentration to 0.25 µM still completely abolished the volley. This experiment demonstrates that the *in vivo* effect of the toxin is not mediated via the TTX-sensitive neurotoxin receptor on voltage-gated sodium channels found within the mammalian central nervous system.

## 3. Experimental Section

### 3.1. L. longissimus Mucus Collection

Specimens of *L. longissimus*, identified by assoc. prof. Malin Strand, were collected in the Koster Fiord on the west coast of Sweden (lat 58.927334, long 11.079712), and in Millport, Scotland (lat 55.753586, long −4.928616). No specific permissions were required for these collection activities. Living specimens of approximately 10 g was placed in an Erlenmeyer flask containing 50 mL of sea water. Mucus production was stimulated via tactile disturbance, and the released mucus mixture was subsequently sampled into a syringe and sterile filtrated (0.20 μm), rendering the mucus fraction used in further toxicity tests and in *Vibrio alginolyticus* cultivation described below.

### 3.2. Shore Crab Bioassay of TTX and L. longissimus Mucus

To assess the toxic effects at various stages of the analysis process, a simple bioassay was carried out on shore crab (*Carcinus maenas*), in a manner similar to the protocol used by Kem [26]. For the bioassays performed on shore crabs no permissions were required. Typically, 0.5 mL of sample (mucus or fraction) was injected into the shore crab branchial chamber via the soft tissue surrounding the protopod base of the third periopod. Crab weight typically in the range 20–30 g. For toxic fractions, this led to tremor followed by complete paralysis. For control purposes, TTX (Sigma-Aldrich, St. Louis, MO, USA) (0.5 mL of concentrations of 0.04, 0.4, 4 μg/mL) dissolved in sea water, was injected as described above.

### 3.3. Bacterial Growth and Identification

Thiosulfate-citrate-bile salts-sucrose (TCBS) agar was used to cultivate any *Vibrio* bacteria from the crude (unfiltered) mucus from a living specimen of L. longissimus. The TCBS agar is highly selective for Vibrio species [27]. A small amount of mucus, directly sampled from the epidermis of *L. longissimus*, was smeared onto the agar according to standard procedures and cultured at 23 °C for 48 h after which yellow colonies (indicating *Vibrio* bacteria) were plated onto Whatman FTA cards and sent for Sanger DNA sequencing of both strands using a Beckman-Coulter Sequenator. Standard nucleotide blast in Genbank (2010) targeting 16S rRNA sequence homology [19] was used for identification. In parallel, the same colonies were transferred to Nutrient Broth (Difco) liquid medium, prepared with sterile filtered sea water. The triplicates of three cultures, sterile sea water (SW), sterile filtered (SF) mucus and autoclaved (A) mucus, were added to control cultures and mucus-infused cultures, respectively, at *t* = 0 h and *t* = 96 h. The cultures were followed for 168 h and sampled every 24 h for optical density measurement at 620 nm (Hitachi U-1100 spectrophotometer, Hitachi, Mountain View, CA, USA). The *Vibrio* culture in liquid medium was sampled at different time points, and the supernatants were collected by centrifugation and filtration. These samples were then subjected to analysis by MS using direct infusion. LC-MS analysis and shore crab assay was performed on all samples.

### 3.4. LC and LC-MS on V. alginolyticus Cultures

Samples of 5 μL TTX standard (Sigma-Aldrich) (10 μg/mL) dissolved in MeCN/NH_4_COOH 50 mM, pH 5.8, (1/1, *v*/*v*) and sea water, respectively, were analyzed on an in-house packed C18-SCX (3:1) column (150 mm × 2.1 mm ID) using Supelco chromatographic material from Sigma-Aldrich. The chromatographic mobile phase (eluent buffer) used was 10 mM NH_4_OAc, pH 4/MeOH (90/10, *v*/*v*) on a μLC system (Perkin Elmer, St. Louis, MO, USA) connected to an ESI-Quadropole-TOF (QSTAR^®^ pulsar-i-Q-TOF tandem mass spectrometer (PE Sciex, Toronto, ON, Canada)). The isocratic LC flow rate was set to 0.20 mL/min over the chromatographic column. A 1:1 joined flow of MeOH + 0.15% formic acid resulted in a final flow towards the MS of 0.40 mL/min. Samples were run both in eluent buffer and sea water. The limit of detection (LOD) for the LC-MS method was 0.1 μg/mL and the limit of quantification (LOQ) 0.13 μg/mL. Subsequently, TTX spiked nutrient broth (33% sterile culture media + 67% MeOH + TTX standards to the final concentrations 0, 1.25, 2.5, 5 and 10 μg/mL) were analyzed under the same conditions. Filtered *V. alginolyticus* culture samples (SW, A, SF; *t* = 0, 24, 48 h) were then subject to LC-MS analysis via the same method. In addition, selected culture samples were spiked with TTX as an internal control to a final concentration of 1.25 mg/L. Finally, MS/MS analysis of TTX and the reoccurring similar peak was performed. Shore crab assay was carried out on fractions containing the unidentified compound with *m*/*z* 320.08.

### 3.5. Attempted TTX-Enrichment from Mucus of L. longissimus

In an attempt to enrich TTX, a large-scale model was developed (first validated using a TTX reference): 245 mL of filtrated (0.20 μm) mucus collected from *L. longissimus* was passed through a chromatography column filled with 19.3 g Lichroprep RP-8 40–63 μm. This was followed by two washing steps (5 × 30 mL·H_2_O and 5 × 50 mL·MeOH) and subsequent extraction of the bound fraction using 3 × 75 mL H_2_O/MeOH/HOAc, 67/28/5 (*v*/*v*). Fractions of this extract were analyzed using LC-MS and, after pH neutralization using NaOH, tested in the shore crab assay.

### 3.6. Analysis of Potential TTX-Macromolecular Association

First 2 mL mucus from *L. longissimus* spiked with bovine serum albumin (BSA) (Sigma-Aldrich) to a final concentration of 0.1 mg/mL was loaded onto a gel filtration column (Superdex 200, prep grade) at a flow rate of 0.5 mL/min, using 0.05 M ammonium bicarbonate, pH 7.8, as the eluent. Absorbance was followed at 280 nm using a Hitachi U-1100 spectrophotometer. Fractions were collected and tested in the shore crab assay. Reference analyses using only mucus or BSA were performed analogously.

### 3.7. Boronate-Affinity Chromatography of L. longissimus Mucus

First, gel filtration was carried out using the same setup as above, using 2 mL mucus from *L. longissimus* loaded onto the gel filtration column at a flow rate of 0.8 mL/min, using 0.05 M ammonium bicarbonate, pH 7.8, as eluent. The fractions collected were then separated on a TSKgel Boronate-5-PW (7.5 mm × 7.5 cm) column using the same HPLC-MS system as previously described. The solvent system was constructed using the same aquatic buffer as for the gel filtration experiment above, with a post column 1:1 join flow of pure MeOH (final flow rate 1 mL/min). Prior to the MS entrance, the flow was split 1:10. Duplicate injections were performed on all toxic fractions (as assessed via shore crab assay): 200 μL was injected and an MS trace was captured on the eluent. Based on this, a second injection was carried out and used for fraction collection. These fractions were then used in the shore crab assay.

### 3.8. Size Fractionation of L. longissimus Mucus

Sterile filtered (0.2 μm) *L. longissimus* mucus was filter-fractionated using successively smaller filters: 10 kDa, 5 kDa, 1 kDa; 10 kD (Amicon Pro, Merck Millipore, Darmstadt, Germany), 5 kD (Vivaspin 20, Vivaproducts, Littleton, MA, USA), 1 kD (Microsep 1K, Pall, Cortland, NY, USA), rendering fractions of approximately <10 kDa; <5 kDa and >1 kDa, which were loaded onto the gel filtration column. Fractions were collected and tested in the shore crab assay.

### 3.9. Preparation of Rat Hippocampal Slices

Electrophysiological experiments were performed on hippocampal slices from 40- to 50-day-old male Wistar rats (in-house breeding). The animals were euthanized according to the guidelines of the ethical committee for animal research. Rats were anesthetized by inhalation of isoflurane (Abbott, Abbott Park, IL, USA; 1 mL of isoflurane in a 5 L box until the limb withdrawal reflex was removed) and then decapitated. The brain was rapidly removed and placed in an ice-cold solution containing (in mM): 140 choline-Cl, 2.5 KCl, 0.5 CaCl_2_, 7 MgCl_2_, 25 NaHCO_3_, 1.25 NaH_2_PO_4_, 0.5 ascorbic acid, and 7 d-glucose. Transverse hippocampal slices (400 μm thick) were cut with a vibratome (Microm HM650, Microm International GmbH, Walldorf, Germany) in the same ice-cold solution, and subsequently stored in a solution containing (in mM): 124 NaCl, 3 KCl, 2 CaCl_2_, 4 MgCl_2_, 26 NaHCO_3_, 1.25 NaH_2_PO_4_, 0.5 ascorbic acid, 3 myo-inositol, 4 d,l-lactic acid, and 10 d-glucose at 25 °C. After approximately 60 min of storage, a single slice was transferred to a recording chamber where it was kept submerged in a constant flow (~4.4 mL/min) at 30–32 °C. During experiments the perfusion solution contained (in mM): 124 NaCl, 3 KCl, 2 CaCl_2_, 1 MgCl_2_, 26 NaHCO_3_, 1.25 NaH_2_PO_4_, and 10 d-glucose. All solutions were continuously bubbled with 95% O_2_ and 5% CO_2_ to get a pH at 7.3–7.4.

### 3.10. Axonal Conductance Recording and Analysis

The efficiency of the nemertea-derived toxin in blocking voltage-gated sodium channels was tested by stimulating the Schaffer collateral fibers of the hippocampus, and measuring the axonal conductance in the stratum radiatum. Doses of the unknown toxin (based on shore crab assay) were added to the perfusion in amounts corresponding to a concentration of 12× the dose that is commonly used to block axonal conductance in rats [28]. Every experiment was ended by adding 0.25–0.5 µM of commercially available TTX (Sigma-Aldrich, Darmstadt, Germany). Extracellular fields (fEPSPs) were recorded using a pipette with 1 M NaCl placed in the stratum radiatum, Sampling frequency was set to 10 kHz, and filtered at 3 kHz, by means of an EPC-10 amplifier (HEKA Elektronik, Lambrecht, Germany). Schaffer collateral/commissural afferents were stimulated using 0.2 ms biphasic (negative/positive) constant current pulses (5–20 µA; STG 1002, Multi Channel Systems, Reutlingen, Germany) delivered through an insulated tungsten microelectrode (resistance ~ 0.3–0.5 MΩ). Stimulation electrodes were positioned in the stratum radiatum and synaptic inputs received test pulse stimulation every 5 s. Evoked responses were analyzed off-line using custom-made IGOR Pro (WaveMetrics, Lake Oswego, OR, USA) software. Data is expressed as means ± SEM. Statistical significance for independent samples was evaluated using paired *t*-test or Student’s *t-*test, unless otherwise is indicated.

All rat slice experiments were performed at the Department of Biological and Environmental Science at the University of Gothenburg and covered by permit 202/2010 from the Animal Ethics Committee, Gothenburg. All surgery was performed under sodium pentobarbital anesthesia, and all efforts were made to minimize suffering. The Animal Ethics Committee specifically approved this study.

## 4. Conclusions

The mucus of *L. longissimus* is highly toxic, as assessed through injections of this mucus into the branchial chamber of *Carcinus maenas*. Numerous reports have indicated the presence of TTX in *Vibrio* bacteria [10,20,21,29,30,31,32,33]. This led us to hypothesize that finding these bacteria in the mucus would indicate the presence of TTX, and thus explain the toxicity. The presence of *Vibrio alginolyticus* was indeed demonstrated in the mucus of *L. longissimus*. However, after isolation and cultivation of the bacteria, we were unable to find any traces of TTX, despite substantial efforts. Our conclusion is that, under the conditions employed in this study, *Vibrio alginolyticus*, neither alone, nor when infused with the mucus of *L. longissimus*, produces TTX. If so, TTX is not produced in concentrations above the level of detection of the analytical LC-MS method, nor in any concentrations high enough that can explain the effect in the shore crab assay. This conclusion supports the observations of Carroll *et al.* [10], who could not observe TTX production after the first generation in a similar system. It may be questioned if TTX was ever observed in their study, as the methods employed cannot detect TTX conclusively.

The mucus of *L. longissimus* did, however, produce a toxic effect apparently on par with that of TTX, but from our analyses it can be concluded that TTX is not the origin of the toxic effect, as it was not present in the mucus. Size fractionation and gel filtration experiments indicated the presence of toxin/-s with a molecular weight of approximately 5 kDa or less. As the toxic mucus has an apparent mode of action closely resembling that of TTX when tested on *C. maenas*, it would appear reasonable to assume that the mucus toxin acts via the same mechanism (voltage-gated sodium channels). However, our results on hippocampal slices from Wistar rats demonstrate clearly that the effect is unrelated to the TTX receptor. The toxin is thus acting through a different mechanism. Further investigation into the chemistry of the mucus toxin was outside the scope of this study. For future studies it may prove rewarding to seek molecules larger than TTX but smaller than 10 kDa. Some reports indeed point in this direction [34,35].

A final observation from our experiments is the need to combine different mass spectrometry techniques, liquid chromatography, and relevant bioassays to reveal the presence, or in this case the absence, of compounds such as TTX. In our opinion, the results in this study, and specifically the TTX-like mass observed, may indicate a need to revisit earlier claims of bacterial TTX production.

This claim is further strengthened by observations from the extensive work by Salvitti *et al.* [36] where 102 bacterial strains were isolated and identified from toxic *Pleurobrancheaea maculata* and *Stylochoplana* sp. Despite significant culturing efforts, no evidence of TTX production could be observed. In addition, based on the literature review of 25 reports of bacterial production of TTX included in their paper, all but one, where LC-MS evidence of TTX itself is provided [33], rely on indirect observations.

In conclusion, the mucus of *Lineus longissimus* is highly toxic as assessed by an *in vivo* shore crab assay. Our study shows that this toxin (if only one compound) is not TTX and does not act via the TTX receptor. In addition, our results imply that TTX may have been erroneously identified in some studies of this kind as a result of inadequate evidence. This should warrant a reinvestigation of several published claims to confirm or disprove TTX presence in different species.

## Figures and Tables

**Table 1:** Nemertean species reported to be involved in synthesis of TTX and derivatives.

Class	Order	Species	Source
Anopla	Palaeonemertea	*Cephalothrix* sp.	[11,12,13]
		*Cephalothrix linearis*	[14,15]
		*Cephalothrix rufifrons*	[10]
		*Cephalothrix simula*	[16,17]
		*Tubulanus punctatus*	[9]
	Heteronemertea	*Lineus alborostratus*	[16]
		*Lineus fuscoviridis*	[9]
		*Lineus longissimus*	[10]
		*Lineus ruber*	[10]
		*Lineus torquatus*	[16]
		*Lineus viridis*	[10]
		*Ramphogordius sanguineus*	[10]
		*Riseriellus occultus*	[10]
Enopla	Hoplonemertea	*Amphiporus* sp.	[16]
		*Amphiporus lactifloreus*	[10]
